# Cross-Subject Registration-Based Augmentation: Alleviating Anatomical Misalignment in Trauma CT for Robust Hemorrhage Segmentation

**DOI:** 10.3390/diagnostics16142154

**Published:** 2026-07-09

**Authors:** Sujong Shin, Jaewoo Chung, Jungchan Cho, Sang-Il Choi

**Affiliations:** 1 Department of AI-Based Convergence, Dankook University, Yongin 16890, Gyeonggi-do, Republic of Korea; shinsjn@dankook.ac.kr; 2 Department of Neurosurgery, Dankook University College of Medicine, Cheonan 31116, Chungcheongnam-do, Republic of Korea; j.chung@dankook.ac.kr; 3 Department of Computing, Gachon University, Seongnam 13120, Gyeonggi-do, Republic of Korea; 4 Department of Computer Engineering, Dankook University, Yongin 16890, Gyeonggi-do, Republic of Korea

**Keywords:** traumatic brain injury, intracranial hemorrhage segmentation, computed tomography, data augmentation, anatomical registration

## Abstract

**Background/Objectives:** In emergency settings, it is often infeasible to place patients in an anatomical position for CT scanning. Consequently, emergency brain CT scans of patients with traumatic brain injury frequently demonstrate considerable anatomical misalignment. These inconsistencies compromise the performance of deep learning-based 3D hematoma segmentation. This study aims to enhance segmentation robustness by proposing a registration-based data augmentation strategy utilizing anatomical landmarks. **Methods:** We propose a framework termed cross-subject registration-based augmentation (CSRA), which uses anatomical landmarks to rigidly register patient CT volumes to selected reference CT volumes and generate anatomically aligned CT-label pairs for training. **Results:** A total of 339 patients who underwent brain CT imaging were enrolled from a Level 1 trauma center. CSRA-3s achieved the highest mean Dice and IoU and the lowest mean HD95 among the evaluated augmentation strategies. Patient-level paired analysis showed that the most robust statistically supported benefit was a significant reduction in HD95 compared with a conventional geometric augmentation baseline, indicating improved boundary agreement. **Conclusions:** The proposed augmentation strategy mitigated the effect of anatomic position discrepancies on segmentation performance, particularly in terms of boundary agreement, without modifying existing model architectures. CSRA may serve as a model-agnostic training-time augmentation strategy for improving segmentation robustness in anatomically inconsistent emergency CT imaging, although multicenter external validation is required before clinical deployment.

## 1. Introduction

More than 20 million cases of traumatic brain injury (TBI) occur worldwide annually, with approximately 1.7 million cases reported annually in the United States [1,2,3]. In Korea, the annual incidence of TBI is estimated to be approximately 79.4 per 100,000 population [4]. In 2021, approximately 70,000 TBI-related deaths were reported in the United States, and trauma remains one of the leading causes of mortality in Korea [5].

Initial brain computed tomography (CT) plays a pivotal role in the acute evaluation and prognostic stratification of TBI and serves as the basis for scoring systems that incorporate intracranial hemorrhage characteristics, including type, volume, and location [6,7,8,9]. In recent years, deep learning-based intracranial hemorrhage segmentation on non-contrast head CT has progressed from single-target hematoma segmentation to more complex settings, including separate segmentation of intracerebral and intraventricular hemorrhage, perihematomal edema segmentation, quality assessment, uncertainty-aware segmentation, and multi-task acute hemorrhage analysis [10,11,12,13,14]. Despite these advances, robustness under heterogeneous acquisition conditions remains an important challenge, particularly in emergency trauma CT where non-ideal head positioning, anisotropic resolution, and variable patient anatomy can degrade model performance [15,16].

In TBI, intracranial hemorrhage often presents with heterogeneous lesion stages and hemorrhage types and may be accompanied by traumatic structural abnormalities, including compound, comminuted, depressed, or blowout fractures. These structural alterations introduce registration errors, thereby degrading the performance of the deep learning models during image analysis. These variations may be difficult to model sufficiently using conventional augmentation techniques alone [17]. Moreover, in emergency CT acquisition, patient discomfort or impaired consciousness often prevents maintenance of an ideal anatomic position, and console-based post-acquisition realignment is limited and operator-dependent. Once CT images are reconstructed and stored as DICOM files, scanner-console-based re-reconstruction or raw-data-based realignment may no longer be available; therefore, image-domain strategies are needed to mitigate pose-related variability in downstream analysis.

**Figure 1:**
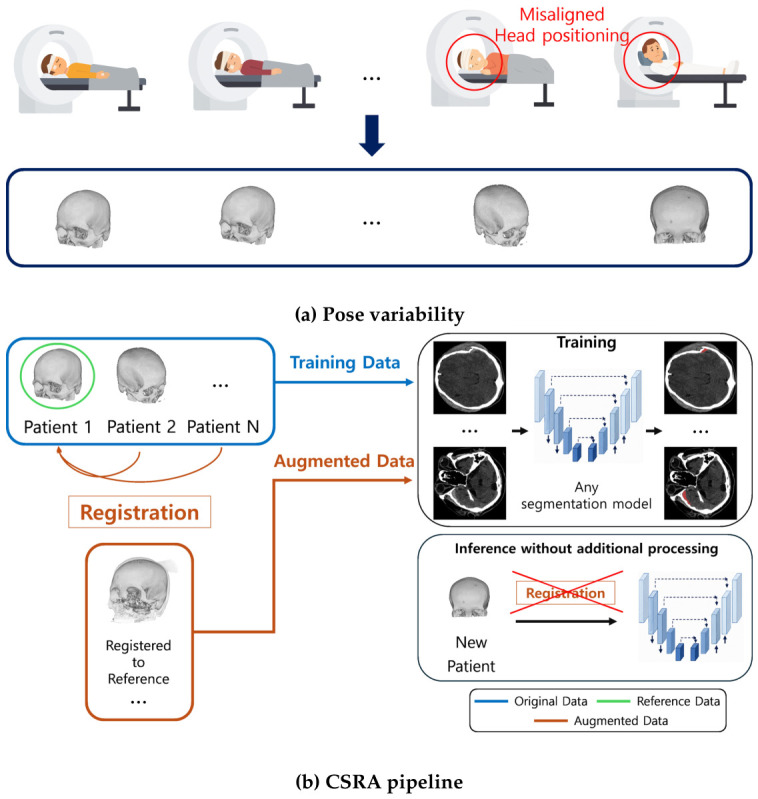
Overview of the proposed CSRA framework. (**a**) Inter-subject pose variability. (**b**) CSRA-based segmentation pipeline.

In this context, several anatomy-aware approaches, including atlas-based, registration-driven, and landmark-guided methods, have been proposed to mitigate anatomical misalignment by improving anatomical correspondence and structural consistency [18,19,20,21,22,23,24,25,26,27,28,29,30,31]. Test-time augmentation methods have also been used to improve robustness or assess prediction variability by evaluating multiple transformed inputs or predictions during inference [32]. However, these approaches do not directly address training-time generation of anatomically aligned CT-label pairs for emergency trauma CT hematoma segmentation while preserving direct inference on original, non-registered CT volumes. To address this gap, we propose cross-subject registration-based augmentation (CSRA), a landmark-guided training-time augmentation strategy for trauma CT hematoma segmentation. Inter-subject pose variability in emergency trauma CT is illustrated in Figure 1a, and an overview of the proposed CSRA framework is shown in Figure 1b.

CSRA is conceptually related to prior registration-based and anatomy-aware approaches because it leverages anatomical correspondence across subjects. However, it differs by using sparse craniofacial and skull landmarks with rigid cross-subject registration to generate anatomically aligned CT-label pairs for training, rather than relying on random geometric perturbations, atlas label propagation, or learned deformable registration networks. This design was intended to compensate for emergency acquisition-related head pose variability while preserving patient-specific hematoma morphology and segmentation label geometry. CSRA is model agnostic and can be integrated into existing deep learning segmentation pipelines without modifying the network architecture. Because registration is confined to training-set construction, CSRA does not require atlas normalization or reference-space registration during standard inference; the trained segmentation model can therefore be applied directly to original emergency CT volumes.

The main contributions of this study are threefold: We propose CSRA for generating anatomically aligned CT-label pairs in trauma CT hematoma segmentation; design it as a training-time, model-agnostic augmentation strategy that preserves direct inference on original emergency CT volumes; and evaluate its segmentation performance, registration robustness, and landmark-based anatomical pose representation against conventional geometry-oriented augmentations.

The remainder of this paper reviews related literature, describes the data and CSRA framework, presents the experimental settings and results, and discusses the implications and limitations of the proposed approach.

## 2. Literature Review

This section summarizes prior studies directly related to hemorrhage segmentation, data augmentation, and anatomy-aware registration, which motivate the proposed CSRA framework.

### 2.1. Conventional Data Augmentation

To improve segmentation robustness, conventional augmentation strategies such as flipping, rotation, affine transformation, elastic or deformable transformation, and intensity perturbation have been widely used in medical image segmentation [17,33,34]. These methods increase training diversity and reduce overfitting by applying image-level transformations [17,33,34]. However, they generally operate within individual images and do not explicitly model anatomical correspondence across different subjects, in contrast to anatomy-aware or registration-driven augmentation strategies [21,22,24]. Therefore, although conventional augmentations can simulate appearance or geometric variability, they may not fully capture inter-subject anatomical correspondence associated with pose misalignment in emergency trauma CT.

### 2.2. Anatomy-Aware and Registration-Based Approaches

Beyond conventional augmentation, several anatomy-aware approaches have addressed inter-subject anatomical pose misalignment by incorporating spatial correspondence more explicitly. Atlas-based and multi-atlas segmentation methods align subjects to a common anatomical space and transfer anatomical priors or labels across subjects [18,19,20]. Registration-driven augmentation and joint registration–segmentation frameworks further demonstrate that anatomically meaningful transformations can improve segmentation robustness by generating realistic spatial variation [21,22,23,24]. Landmark- and keypoint-guided registration methods have also been used for anatomical alignment, registration initialization, and correspondence estimation in brain and head CT imaging [25,26,27,28,29,30,31]. These studies indicate that registration and anatomical correspondence are valuable for improving structural consistency. Test-time augmentation methods represent another related strategy in which multiple transformed inputs or predictions are evaluated during inference to improve robustness or assess prediction variability in medical image segmentation [32]. However, most existing approaches focus on atlas normalization, deformable registration, learned registration networks, inference-time augmentation, or registration itself, rather than on generating training-time CT-label pairs for emergency trauma CT hematoma segmentation. Therefore, a practical training-time augmentation strategy is needed to address inter-subject pose misalignment while preserving direct inference on original, non-registered emergency CT volumes.

## 3. Methods

### 3.1. Problem Formulation

**Figure 2:**
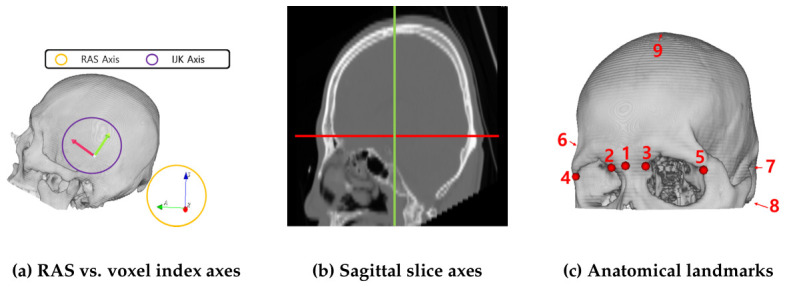
Coordinate systems and anatomical landmarks used in CSRA. (**a**) Comparison between the global anatomical RAS coordinate system and the voxel index coordinate axes (IJK). (**b**) Sagittal slice view illustrating voxel-defined axes, with red and green lines indicating axial and coronal directions, respectively. (**c**) Nine anatomical landmarks used for landmark-based rigid registration: 1, nasion; 2 and 3, junctions of the orbital rim and frontomaxillary suture; 4 and 5, junctions of the orbital rim and frontozygomatic suture; 6 and 7, anterior margins of the external auditory canals; 8, opisthion; and 9, vertex.

CT volumes are defined within a global anatomical coordinate system such as RAS, but individual acquisitions often exhibit substantial orientation differences due to variations in imaging environments and patient positioning. Specifically, as illustrated in Figure 2a, the discrete voxel grid is defined by image index coordinates, commonly denoted as IJK axes in 3D Slicer terminology. These voxel index axes may differ from the patient-based anatomical RAS coordinate system, creating discrepancies between anatomical orientation and voxel representation [35,36]. This mismatch is evident in the sagittal slice visualization in Figure 2b, where the displayed axial and orthogonal axes are governed by voxel grid orientation rather than fixed anatomical directions. Variations in head positioning, particularly under emergency acquisition conditions, further introduce structural inconsistency across subjects, complicating anatomical correspondence and reducing the robustness of deep learning feature extraction.

To address this problem, we propose CSRA as a landmark-guided training-time augmentation strategy and define nine anatomical landmarks for estimating cross-subject rigid transformations, as shown in Figure 2c. These nine landmarks were selected because they are distributed across the anterior facial skeleton, cranial base, and superior cranial extent, thereby providing non-collinear spatial constraints for estimating a stable rigid transformation. Their broad anatomical distribution helps constrain rotation and translation in three-dimensional space, while relying on structures that are generally less affected by hematoma morphology than intracranial lesion boundaries [26,27,28,29].

### 3.2. Landmark-Guided Training-Time Augmentation

CSRA estimates rigid transformations from anatomical landmarks to preserve lesion geometry during augmentation. Unlike affine or deformable registration, rigid registration avoids scaling, shearing, and local warping that may alter clinically meaningful lesion geometry. This rigid formulation is suitable for emergency trauma CT, where misalignment frequently arises from patient-positioning differences during acquisition and can be approximated as global translation and rotation.

For each patient, we denote a 3D CT volume as $x_{i}\in R^{H\times W\times D}$ and its corresponding hemorrhage segmentation mask as $y_{i}\in {\left\{0,1\right\}}^{H\times W\times D}$, where *H*, *W*, and *D* represent spatial dimensions. The hematoma segmentation model is formulated as a mapping $f_{\theta }:x_{i}\mapsto {\hat{y}}_{i}$, where ${\hat{y}}_{i}$ denotes the predicted hemorrhage mask [37,38].

Each subject is additionally associated with an ordered, anatomically indexed landmark list $L_{i}=\left(p_{i}^{1},\ldots ,p_{i}^{9}\right)$, where each index *k* corresponds to a predefined anatomical landmark and $p_{i}^{k}\in R^{3}$ denotes its voxel coordinate. The landmark detection model $g_{\phi }$, implemented as a three-dimensional HRNet-W32-based volumetric heatmap regression model [39,40], receives preprocessed 3D CT volumes and predicts nine landmark-specific volumetric heatmaps. Subject-specific landmark coordinates ${\hat{L}}_{i}$ are obtained from the predicted heatmaps using a three-dimensional soft-argmax operation.

Rigid registration is defined between subject *i* and a designated reference subject *r* by estimating a transformation matrix $R_{i\to r}$ that minimizes landmark alignment error, expressed as $R_{i\to r}=\arg\min_{R}\sum_{k}{∥R\left(p_{i}^{k}\right)-p_{r}^{k}∥}^{2}$. The rigid transformation matrices were estimated using Horn’s method [41]. The current implementation used all nine anatomical landmarks for rigid transformation estimation. When landmarks are missing, the same rigid transformation framework can be extended by estimating the transform from available corresponding landmarks, provided that at least three non-collinear landmark pairs are available [41,42]. However, robust missing-landmark handling was not explicitly implemented in the present pipeline and remains a subject for future development.

For clarity of notation in subsequent processing, we denote the registration operation from subject space to the reference space as a CSRA transform operator $T_{i\to r}\left(\cdot \right)$, which is applied to both image and label volumes to produce structurally aligned samples ${\tilde{x}}_{i}=T_{i\to r}\left(x_{i}\right)$ and ${\tilde{y}}_{i}=T_{i\to r}\left(y_{i}\right)$. When predictions or intermediate results need to be expressed back in the original subject space, the corresponding reverse mapping is denoted as $T_{r\to i}\left(\cdot \right)$.

**Figure 3:**
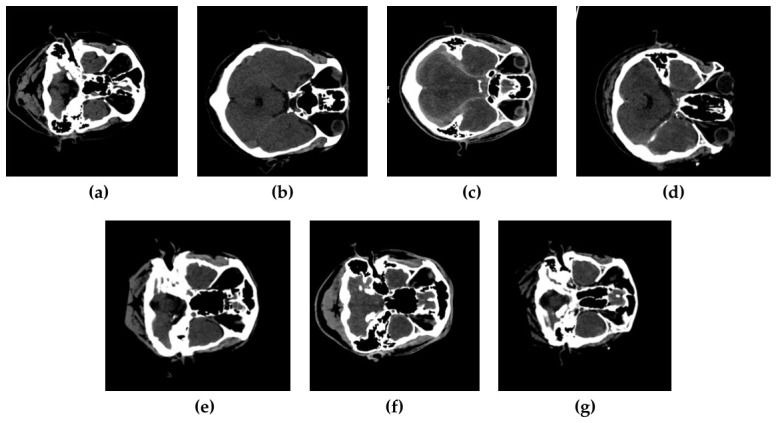
Representative 2D CT slices illustrating the effect of landmark-based rigid registration. Ref. denotes the reference slice, and P1–P3 denote three example patients. (**a**) Ref.; (**b**) P1 before; (**c**) P2 before; (**d**) P3 before; (**e**) P1 after; (**f**) P2 after; (**g**) P3 after.

The effect of this alignment is illustrated in Figure 3. As illustrated in Figure 3b–d, representative 2D slices extracted from CT volumes that originally exhibited inter-patient anatomical variability were structurally aligned through the proposed registration method, yielding registered 2D slices (Figure 3e–g) with anatomical configurations closely resembling the representative reference slice (Figure 3a).

### 3.3. Reference Volume Selection and Training-Set Augmentation

The CSRA procedure consists of three steps: reference-volume selection, landmark-based rigid registration, and training-set augmentation.

#### 3.3.1. Reference Volume Selection

Reference CT volumes representing diverse head orientations were selected using quaternion-derived rotational distances and farthest point sampling [43]. To compute the rotational distance, rigid transformations were first estimated between candidate CT volumes using the corresponding anatomical landmarks. The rotational component of each transformation was converted into a unit quaternion, and the rotational distance between two candidate volumes, $d(q_{a},q_{b})$, was computed as

$$d\left(q_{a},q_{b}\right)=2\cos^{-1}\left(\right|q_{a}^{⊤}q_{b}\left|\right),$$

where $q_{a}$ and $q_{b}$ denote unit quaternions representing the rotational components of two candidate volumes. Based on the resulting pairwise rotational distance matrix, farthest point sampling was applied to select reference volumes with separated anatomical orientations. Reference-volume selection was performed independently within the training set of each cross-validation split to avoid information leakage from the corresponding test fold. As the initial reference, we selected the training-set candidate with the smallest average rotational distance to all other training candidates, corresponding to the medoid of the training-set orientation distribution. Each subsequent reference volume was then selected as the candidate that maximized the minimum rotational distance to the already selected reference set.

#### 3.3.2. Landmark-Based Rigid Registration and Training-Set Augmentation

For each subject-reference pair, a rigid transformation was estimated from the corresponding anatomical landmarks and applied to both the CT volume and the hematoma segmentation mask. Linear interpolation was used for CT volumes, whereas nearest-neighbor interpolation was used for segmentation masks to preserve discrete label values [34,44]. This process generated registered CT-label pairs in the reference space.

Finally, these registered CT-label pairs were added only to the training set as augmented samples. CSRA was applied only to the training data within each cross-validation split, whereas the corresponding test fold was kept unchanged and all primary segmentation evaluations were performed directly in the original image space without atlas alignment or reference-space registration. Reference-based transformations were used only for the optional exploratory prediction variability visualization described below.

#### 3.3.3. CSRA Variants and Comparison

In this study, CSRA-1s, CSRA-2s, and CSRA-3s used one, two, and three reference volumes selected by this procedure, respectively. Rather than directly proving complete pose invariance, this reference-driven alignment was intended to expose the segmentation model to anatomically meaningful pose variation while preserving structural correspondence across subjects, thereby improving robustness to anatomical pose variability.

For comparison, conventional augmentation strategies apply stochastic operators T∈T such as rotation, affine transformation, or elastic deformation to generate perturbed samples ${\hat{x}}_{i}=T\left(x_{i}\right)$. While these transformations simulate geometric variability, they do not explicitly enforce anatomical correspondence across subjects. In contrast, CSRA introduces landmark-constrained transformations that preserve inter-subject structural alignment while expanding anatomically meaningful diversity arising from both intrinsic patient anatomy and extrinsic acquisition-related factors.

## 4. Experimental Results and Discussion

### 4.1. Study Cohort and Data Splitting

This retrospective study was approved by the Institutional Review Board (IRB No. 2023-12-009), and the requirement for informed consent was waived because of the retrospective study design. Patients older than 18 years who underwent non-contrast brain CT for acute TBI at the Level 1 Trauma Center of our institution from January 2019 onward and had CT-visible traumatic intracranial hemorrhage requiring manual segmentation were eligible for inclusion. Cases without a clear CT-visible hemorrhage target, including isolated concussion and non-hemorrhagic diffuse axonal injury, were not included because they did not provide a reliable lesion mask for supervised hemorrhage segmentation. CT scans were excluded when severe motion artifacts produced discontinuity or distortion of the skull contour over two or three consecutive slices without a corresponding skull fracture; these cases were additionally reviewed using 3D skull-bone reconstruction to confirm abnormal skull-contour deformation. DICOM studies were also excluded when essential spatial metadata, such as inter-slice distance, were missing or when the image series could not be retrieved from the institutional DICOM server. After applying these criteria, 339 patients were included in the final analysis.

Each of the 339 patients contributed one 3D brain CT volume and its corresponding hematoma segmentation annotation. Each 3D CT volume was assigned to a single fold, and no patient-level data were shared across the training and test sets. The final model performance was assessed using five-fold cross-validation with patient-wise data splitting, and each augmentation strategy was evaluated using the same folds to ensure paired comparison across methods.

### 4.2. Image Preprocessing and Annotation

#### 4.2.1. CT Preprocessing

To correct for anisotropic resolution and ensure spatial consistency across subjects, all CT volumes and corresponding annotations were resampled to 0.5×0.5×2.5 mm using identical output spacing and image geometry. Linear interpolation was used for CT volumes, whereas nearest-neighbor interpolation was used for segmentation annotations to preserve discrete label values. For hematoma segmentation, voxel intensity values were clipped to the range of 0–140 Hounsfield Units (HU).

#### 4.2.2. Hematoma Segmentation Annotation

Hematoma regions were annotated through a consensus-based procedure by two neurosurgery specialists with 13 and 2 years of clinical experience, respectively. Rather than generating independent final masks, the specialists iteratively reviewed, cross-checked, and discussed the annotations, jointly correcting ambiguous boundaries, particularly between adjacent SAH and SDH components or within diffuse parenchymal injury, until consensus was reached. The consensus-corrected mask was used as the final ground truth for supervised segmentation. Formal inter-rater agreement was not calculated because the final labels were generated through joint consensus review rather than independent blinded annotation, and because traumatic hemorrhage often presents with intrinsically ambiguous boundaries on CT.

All traumatic hemorrhagic lesions, including epidural hematoma, subdural hematoma, subarachnoid hemorrhage, intraparenchymal hemorrhage/contusion, intraventricular hemorrhage, and diffuse parenchymal injury, were annotated as a single binary hemorrhage class because severe traumatic intracranial hemorrhage frequently contains overlapping components with indistinct boundaries on emergency CT. This binary-label strategy was intended to improve annotation consistency and segmentation robustness in a limited dataset, but it precluded subtype-specific analysis and could not identify subtype-specific pathophysiological mechanisms or associations with clinical outcomes, including mortality.

#### 4.2.3. Landmark Annotation and Heatmap Generation

For landmark detection, CT volumes were clipped to an osseous window of −200 to 2000 HU, linearly normalized to 0–1, and resized to 288×288×64 voxels before model input. Anatomical landmark annotations were generated for each volume using 3D Slicer version 5.6.2 and the Fiducial Wizard extension [45]. Landmark placement was performed by two trained annotators under neurosurgery specialist supervision using a predefined nine-landmark protocol corresponding to the anatomical landmarks shown in Figure 2c. After initial placement, the annotators cross-checked each other’s landmark sets, and the specialist reviewed all cases and corrected inaccurate or inconsistent landmarks when necessary. The average time required for landmark annotation was approximately 40 s per case. Formal inter-rater variability was not calculated because the final landmark labels were generated after cross-checking and specialist correction rather than independent blinded annotation. For network supervision, Gaussian volumetric heatmaps were generated for each of the nine anatomical landmarks and concatenated into a nine-channel heatmap label.

### 4.3. Implementation Details

In this study, a 3D U-Net [37] was adopted as the baseline backbone for volumetric hematoma segmentation [38]. The model received one-channel CT volumes as input and was trained using 256×256×32 voxel patches sampled by positive–negative label-based random cropping with eight samples per volume; the same patch size was used for sliding-window inference. Training was performed for 300 epochs with a batch size of 1, which was the maximum feasible size under GPU memory constraints, using AdamW [46] with a learning rate of $1\times 10^{-3}$ and Dice Focal Loss [47,48]; early stopping was not applied. The CSRA training-set construction followed the procedure described in the Methods Section.

For landmark detection, a three-dimensional HRNet-W32-based volumetric heatmap regression model [39,40] received one-channel 3D CT volumes and predicted nine landmark-specific volumetric heatmaps. Predicted heatmaps were converted into coordinates using a three-dimensional soft-argmax operation with a temperature of 0.5. The model was trained with a batch size of 1 using AdamW with a learning rate of $1\times 10^{-4}$ for up to 3000 epochs, with early stopping after epoch 1000 using a patience of 10 epochs based on validation performance. The training objective was the L1 loss between the soft-argmax-derived coordinates and the ground-truth landmark coordinates.

For the predicted-landmark CSRA experiment, reported below as CSRA-3s*, landmark prediction was performed fold-wise using the same patient-wise cross-validation splits as the segmentation experiments. In each split, the landmark detector was trained without cases from the corresponding segmentation test fold, and only predicted landmark coordinates were used to construct CSRA-augmented training samples. Manual landmark annotations from the segmentation test fold were not used for reference selection, transformation estimation, or training-set augmentation. All experiments were implemented in Python version 3.10.15 using PyTorch version 2.5.1, TorchIO version 0.20.3, and SimpleITK version 2.4.0. Experiments were performed on a workstation equipped with an NVIDIA RTX 3090 GPU (NVIDIA Corporation, Santa Clara, CA, USA).

### 4.4. Augmentation Comparison

#### 4.4.1. Evaluation Metrics

Segmentation performance was evaluated using Dice similarity coefficient, intersection over union (IoU), 95th percentile Hausdorff distance (HD95), sensitivity, and specificity [49]. Dice and IoU assessed volumetric overlap, HD95 assessed boundary agreement, and sensitivity and specificity assessed voxel-level classification; higher Dice, IoU, sensitivity, and specificity and lower HD95 indicate better performance. Metrics were computed for each test fold and reported as mean ± standard deviation across five-fold cross-validation. Patient-level paired comparisons were performed using the Wilcoxon signed-rank test with Holm correction, and 95% paired bootstrap confidence intervals were calculated for the main performance differences.

#### 4.4.2. Compared Augmentation Strategies

Table 1 compares hematoma segmentation performance across existing augmentation baselines and CSRA variants. We selected existing augmentation baselines commonly used in medical image segmentation and related to spatial, resolution, or CT-intensity variation, including Flip RAS, Affine R, Affine RAS, deformable augmentation, random anisotropy, and CT window jitter [17,33,34,50,51,52,53]. Flip RAS introduced axis-wise mirroring; Affine R and Affine RAS simulated rotational variability around the R-axis or across all RAS axes, respectively; and deformable augmentation simulated localized anatomical variation. Flip RAS, Affine R, Affine RAS, and deformable augmentation were chosen as spatial augmentation baselines because they produce anatomically plausible variations without synthetic image content and are directly related to CSRA in terms of geometric variation [33,34,54]. Random anisotropy and CT window jitter were additionally included to evaluate augmentation strategies targeting slice-direction resolution variability and CT-intensity window variation, respectively [51,52,53].

Flip RAS independently flipped CT volumes along each RAS axis with a probability of 0.5. Affine R applied single-axis rotation around the R-axis within ±30°, whereas Affine RAS applied random rotations across all RAS axes within ±30° per axis; scaling was not applied. Deformable augmentation used random 3D elastic deformation with a probability of 0.5, a sigma range of 4–6, a magnitude range of 50–100, and reflection padding. Random anisotropy simulated through-plane resolution variability by downsampling and resampling the CT volume and corresponding segmentation mask along the slice direction. CT window jitter randomly perturbed the HU clipping window around the default 0–140 HU range during training; this intensity augmentation was applied only to CT volumes and not to segmentation masks. For all spatial augmentations, the same spatial transformation was applied to the CT volume and segmentation annotation, using linear interpolation for CT volumes and nearest-neighbor interpolation for masks. CSRA was evaluated using one, two, and three reference volumes, corresponding to CSRA-1s, CSRA-2s, and CSRA-3s, respectively, serving as a partial ablation of the number of reference subjects. CSRA-3s* was additionally included to assess the feasibility of replacing annotated landmarks with predicted landmark locations in a more automated CSRA pipeline.

#### 4.4.3. Segmentation Performance Comparison

As summarized in Table 1, the existing augmentation baselines produced heterogeneous effects across metrics. Flip RAS did not improve mean Dice over the default baseline, suggesting that mirroring increased data diversity but did not address cross-subject anatomical pose misalignment. Affine R produced a small improvement consistent with limited head-tilt variability, whereas Affine RAS and random anisotropy showed the lowest mean Dice values, suggesting that unconstrained multi-axis rotations or slice-direction resolution simulation may introduce variability that does not effectively address cross-subject anatomical pose misalignment. Deformable augmentation, CT window jitter, and random anisotropy produced lower mean Dice than the default baseline, suggesting that local deformation, HU-window perturbation, or slice-direction resolution simulation alone may not sufficiently address cross-subject anatomical pose misalignment.

CSRA-3s achieved the highest mean Dice and IoU and the lowest mean HD95 among all evaluated augmentation settings, although the most robust statistically supported improvement was observed in HD95. Unlike conventional augmentations, CSRA generates augmented samples through landmark-based cross-subject registration, exposing the model to anatomically meaningful pose variation while preserving hematoma geometry through rigid transformation [21,22,24,26]. Compared with Affine R, CSRA-3s significantly reduced HD95 by 8.93 mm (95% paired bootstrap CI: −13.98 to −3.94; Holm-corrected Wilcoxon signed-rank test, p=0.0073), indicating improved boundary agreement between predicted and ground-truth hematoma masks. Dice and IoU showed small positive trends but did not remain significant after multiple-comparison correction; therefore, the main supported benefit of CSRA-3s was improved boundary agreement rather than a large volumetric overlap gain.

The comparison across CSRA-1s, CSRA-2s, and CSRA-3s evaluated the effect of reference-pose coverage. Using multiple reference volumes may reduce dependence on a single reference configuration and expose the model to a broader range of anatomically aligned pose anchors during training, conceptually resembling the use of multiple anatomical references in atlas-based segmentation [18,19]. CSRA-3s* showed a small decrease in overlap-based metrics compared with annotation-based CSRA-3s, although HD95 degradation was more pronounced, indicating that predicted landmarks remain feasible but may affect boundary agreement. After converting the soft-argmax-derived normalized coordinates back to physical image coordinates using the corresponding voxel spacing, the landmark detector achieved a five-fold mean Euclidean localization error of 0.4537 mm across nine landmarks.

### 4.5. Registration and Landmark Robustness Analysis

Anatomical alignment was quantitatively assessed using target registration error (TRE), defined as the Euclidean distance between transformed subject landmarks and corresponding reference landmarks [42]. Across 1014 subject-reference pairs, the post-registration mean, median, and 95th percentile TRE were 6.916, 5.839, and 15.465 mm, respectively, indicating consistent anatomical alignment in the reference space.

Landmark-number robustness was evaluated by estimating rigid transformations using 3, 5, 7, or 9 landmarks and computing TRE on all nine landmarks. The mean TRE decreased from 10.406 mm with three landmarks to 7.620, 7.164, and 6.916 mm with five, seven, and nine landmarks, respectively, while the 95th percentile TRE decreased from 29.313 to 15.465 mm. Thus, using all nine landmarks provided the most stable alignment and reduced large registration errors caused by landmark subset selection.

CSRA was performed offline; excluding file writing, rigid transform estimation and CT-label resampling required 0.177±0.031 s per subject-reference pair, corresponding to approximately 0.18, 0.35, and 0.53 s per subject for CSRA-1s, CSRA-2s, and CSRA-3s, respectively. Therefore, the main computational burden was increased training-set size and storage rather than inference-time computation.

This landmark-number analysis supports the use of all nine landmarks but should be interpreted as a robustness analysis rather than an exhaustive anatomical landmark-group ablation. Future work should evaluate specific landmark groups and compare rigid, affine, and deformable CSRA variants under lesion-geometry-preserving constraints.

### 4.6. Qualitative Evaluation

**Figure 4:**
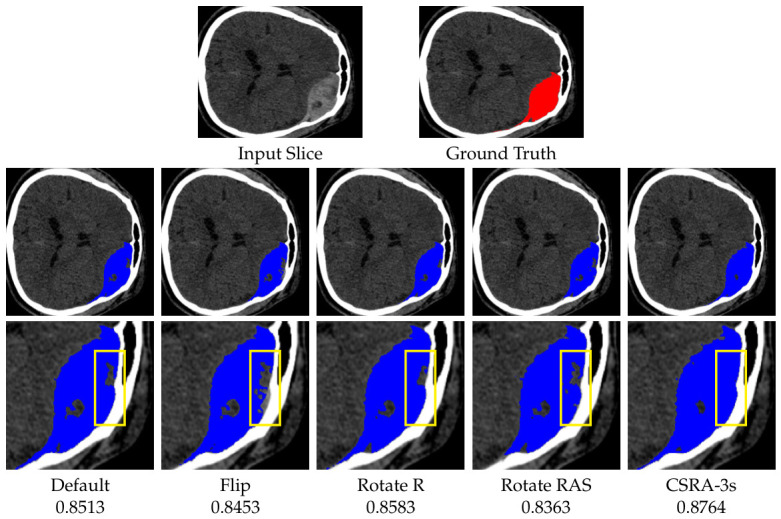
Qualitative examples of segmentation results for patient 342 under different augmentation methods. Blue regions indicate predicted hemorrhage masks, red regions indicate ground-truth (GT) hemorrhage regions, and yellow boxes indicate the magnified regions shown in the zoomed views. Numbers indicate Dice scores.

Figure 4 shows a representative patient case. In the zoomed view, CSRA-3s appears to delineate the hemorrhage boundary more continuously, producing a segmentation that is visually closer to the ground-truth annotation in this representative case. In contrast, conventional transformation-based augmentation methods, such as Flip RAS and Affine R, produced more fragmented and less continuous boundaries in this example. These qualitative findings are consistent with the HD95 analysis and suggest that CSRA may improve boundary continuity and anatomical alignment of the predicted hematoma mask compared with conventional geometric augmentations.

This qualitative pattern may be related to the CSRA design, which integrates inter-patient anatomical variability while preserving structural consistency. Rather than relying solely on subject-independent stochastic geometric transformations, CSRA incorporates landmark-constrained cross-subject alignment, enabling the model to learn structural patterns that better reflect anatomical pose variability.

### 4.7. Effect of CSRA on Low-Dimensional Representation

For further investigation, we visualized the training and test samples in a low-dimensional space using principal component analysis (PCA) [55]. PCA was selected because the purpose of this analysis was to visualize the dominant global geometric variation in the landmark-coordinate space rather than to identify nonlinear latent clusters. Nonlinear methods such as t-SNE or UMAP can be useful for local neighborhood visualization, but their global distances and cluster geometry can be sensitive to hyperparameters and may be less directly interpretable for reference-based geometric analysis. Because CSRA is driven by landmark-based rigid registration, we applied PCA to the landmark vectors used in the registration process, rather than to image-intensity features, to isolate anatomical pose variation from intensity or lesion-appearance variation.

**Figure 5:**
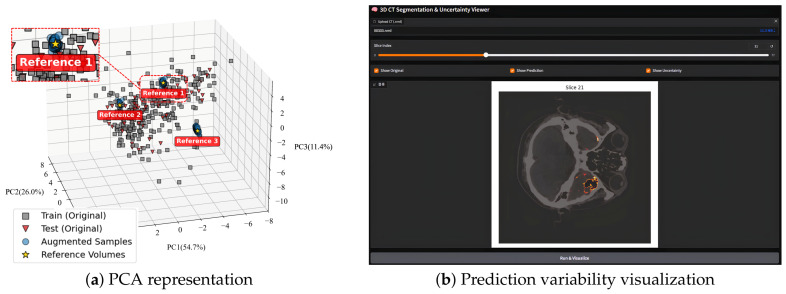
PCA-based visualization of CSRA-augmented samples and example prediction variability visualization interface. (**a**) Three-dimensional landmark-based PCA projection of original training samples, original test samples, CSRA-augmented samples, and selected reference volumes. The axes correspond to PC1, PC2, and PC3, explaining 54.7%, 26.0%, and 11.4% of the landmark-space variance, respectively, with a cumulative explained variance of 92.0%. Enlarged dashed regions highlight representative reference-centered areas in the PCA space for qualitative visualization of CSRA-induced geometric variation. (**b**) Example interface for visualizing CT slices, predicted hemorrhage regions, and qualitative prediction variability maps obtained from multiple reference-based CSRA predictions.

The results are shown in Figure 5a as a three-dimensional PCA projection. PC1, PC2, and PC3 explained 54.7%, 26.0%, and 11.4% of the total landmark-space variance, respectively, corresponding to a cumulative explained variance of 92.0%. In the plot, gray squares indicate original training samples, red triangles indicate original test samples, blue circles indicate CSRA-augmented samples, and yellow stars indicate the selected reference volumes. The enlarged dashed regions highlight representative reference-centered areas, showing that CSRA generates augmented samples around the selected reference volumes in the landmark-based PCA space. This PCA analysis was used as a qualitative visualization of how reference-based registration expands the anatomical pose distribution of the training data, rather than as a formal clustering analysis.

### 4.8. CSRA-Based Prediction Variability Visualization

Although all primary segmentation results in this study were obtained using standard direct inference on the original CT volumes, CSRA can also be used as an optional test-time reference transformation strategy for exploratory prediction variability visualization, following the general principle that test-time augmentation can be used to assess prediction variability in medical image segmentation [32]. Registering an input CT to multiple anatomical references, with $r_{j}$ denoting the *j*th reference, produces structurally diverse predictions ${\hat{y}}_{i}^{\left(r_{j}\right)}=T_{r_{j}\to i}\left(f_{\theta }\left(T_{i\to r_{j}}\left(x_{i}\right)\right)\right)$, which are restored to the original subject space via the inverse transform. An aggregated prediction can be generated from these reference-based outputs, and spatial disagreement among the outputs may provide a qualitative indication of prediction variability. Figure 5b presents an example visualization interface in which the CT slice, predicted hemorrhage region, and prediction variability map can be inspected together. This analysis was intended as an exploratory visualization of potentially unstable regions rather than a fully validated uncertainty quantification framework.

## 5. Limitations

First, the single-center cohort of 339 CT-positive hemorrhagic TBI patients may limit generalizability across scanner vendors, acquisition protocols, emergency workflows, and case severity distributions. Although patient-wise five-fold cross-validation was used for internal evaluation, multicenter external validation is required before clinical deployment. Second, all traumatic hemorrhagic lesions were pooled into a single binary label, preventing subtype-specific segmentation analysis and limiting assessment of subtype-specific associations with clinical outcomes. Third, consensus-corrected segmentation masks and landmark labels were used rather than independent blinded annotations, so formal inter-rater agreement was not calculated; moreover, ambiguous hematoma boundaries and similar CT attenuation between hemorrhage and adjacent structures may introduce annotation uncertainty even after consensus review. Fourth, although CSRA does not add inference-time computation because registration is confined to training-set construction, using multiple reference volumes increases training-set size and storage requirements in proportion to the number of selected references. Finally, CSRA depends on the availability and quality of anatomical landmarks, requiring further evaluation of automated landmark detection, missing-landmark handling, and external landmark-localization robustness. Future work should incorporate multicenter validation, uncertainty-aware training, and multimodal integration of imaging and clinical data to improve clinical utility.

## 6. Conclusions

This study proposed CSRA, a landmark-guided training-time augmentation strategy for robust hemorrhage segmentation in emergency trauma CT. CSRA improved boundary agreement compared with conventional geometric augmentation while preserving direct inference on original CT volumes. These findings suggest that anatomy-aware registration-based augmentation may improve segmentation robustness under acquisition-related pose variability. Multicenter external validation and fully automated landmark handling are required before clinical deployment.

## Figures and Tables

**Table 1:** Segmentation performance comparison between existing augmentation baselines and CSRA variants (mean ± standard deviation, 5-fold cross-validation). * indicates CSRA results generated using predicted landmark locations.

Augmentation	Dice	IoU	HD95	Sensitivity	Specificity
Default	$0.582^{\;\pm 0.038}$	$0.463^{\;\pm 0.029}$	$101.170^{\;\pm 8.046}$	$0.599^{\;\pm 0.037}$	$0.997^{\;\pm 0.000}$
Flip RAS [32]	$0.579^{\;\pm 0.037}$	$0.468^{\;\pm 0.023}$	$102.640^{\;\pm 4.926}$	$0.600^{\;\pm 0.016}$	$0.997^{\;\pm 0.000}$
Affine RAS [32]	$0.550^{\;\pm 0.036}$	$0.460^{\;\pm 0.034}$	$112.630^{\;\pm 8.500}$	$0.562^{\;\pm 0.046}$	$0.997^{\;\pm 0.000}$
Affine R [32]	$0.585^{\;\pm 0.043}$	$0.473^{\;\pm 0.038}$	$108.880^{\;\pm 9.610}$	$0.619^{\;\pm 0.042}$	$0.998^{\;\pm 0.000}$
Deformable [50]	$0.555^{\;\pm 0.043}$	$0.463^{\;\pm 0.038}$	$112.680^{\;\pm 8.810}$	$0.612^{\;\pm 0.039}$	$0.997^{\;\pm 0.000}$
Window jitter [52]	$0.557^{\;\pm 0.034}$	$0.431^{\;\pm 0.041}$	$104.739^{\;\pm 11.002}$	$0.544^{\;\pm 0.037}$	$0.997^{\;\pm 0.001}$
Random anisotropy [34]	$0.549^{\;\pm 0.033}$	$0.426^{\;\pm 0.035}$	$105.480^{\;\pm 10.054}$	$0.535^{\;\pm 0.043}$	$0.998^{\;\pm 0.001}$
CSRA-1s	$0.608^{\;\pm 0.030}$	$0.468^{\;\pm 0.030}$	$103.313^{\;\pm 12.308}$	$0.619^{\;\pm 0.020}$	$0.998^{\;\pm 0.000}$
CSRA-2s	$0.613^{\;\pm 0.031}$	$0.479^{\;\pm 0.040}$	$90.750^{\;\pm 8.677}$	$0.604^{\;\pm 0.048}$	$0.998^{\;\pm 0.000}$
CSRA-3s	$0.616^{\;\pm 0.030}$	$0.481^{\;\pm 0.036}$	$89.735^{\;\pm 8.334}$	$0.624^{\;\pm 0.047}$	$0.998^{\;\pm 0.000}$
CSRA-3s*	$0.608^{\;\pm 0.031}$	$0.465^{\;\pm 0.033}$	$103.565^{\;\pm 8.720}$	$0.611^{\;\pm 0.040}$	$0.998^{\;\pm 0.000}$

## Data Availability

The data presented in this study are available on reasonable request from the corresponding author due to privacy, ethical, and institutional restrictions. The data are not publicly available because they contain patient-level clinical information and were obtained under institutional review board approval. Data sharing may require approval from the relevant institutional review board and/or a data use agreement.
